# e-GRASP: an integrated evolutionary and GRASP resource for exploring disease associations

**DOI:** 10.1186/s12864-016-3088-1

**Published:** 2016-10-17

**Authors:** Sajjad Karim, Hend Fakhri NourEldin, Heba Abusamra, Nada Salem, Elham Alhathli, Joel Dudley, Max Sanderford, Laura B. Scheinfeldt, Sudhir Kumar

**Affiliations:** 1 Center for Excellence in Genome Medicine and Research, King Abdulaziz University, Jeddah, Saudi Arabia; 2 Department of Genetics and Genomic Sciences, Mount Sinai School of Medicine, New York, NY 10029 USA; 3 Institute for Genomics and Evolutionary Medicine, Temple University, Philadelphia, PA 19122 USA; 4 Department of Biology, Temple University, Philadelphia, PA 19122 USA

**Keywords:** Polymorphism, SNP, GWAS, GRASP, Conservation, Disease, Phenotype

## Abstract

**Background:**

Genome-wide association studies (GWAS) have become a mainstay of biological research concerned with discovering genetic variation linked to phenotypic traits and diseases. Both discrete and continuous traits can be analyzed in GWAS to discover associations between single nucleotide polymorphisms (SNPs) and traits of interest. Associations are typically determined by estimating the significance of the statistical relationship between genetic loci and the given trait. However, the prioritization of bona fide, reproducible genetic associations from GWAS results remains a central challenge in identifying genomic loci underlying common complex diseases. Evolutionary-aware meta-analysis of the growing GWAS literature is one way to address this challenge and to advance from association to causation in the discovery of genotype-phenotype relationships.

**Description:**

We have created an evolutionary GWAS resource to enable in-depth query and exploration of published GWAS results. This resource uses the publically available GWAS results annotated in the GRASP2 database. The GRASP2 database includes results from 2082 studies, 177 broad phenotype categories, and ~8.87 million SNP-phenotype associations. For each SNP in e-GRASP, we present information from the GRASP2 database for convenience as well as evolutionary information (e.g., rate and timespan). Users can, therefore, identify not only SNPs with highly significant phenotype-association *P*-values, but also SNPs that are highly replicated and/or occur at evolutionarily conserved sites that are likely to be functionally important. Additionally, we provide an evolutionary-adjusted SNP association ranking (E-rank) that uses cross-species evolutionary conservation scores and population allele frequencies to transform *P*-values in an effort to enhance the discovery of SNPs with a greater probability of biologically meaningful disease associations.

**Conclusion:**

By adding an evolutionary dimension to the GWAS results available in the GRASP2 database, our e-GRASP resource will enable a more effective exploration of SNPs not only by the statistical significance of trait associations, but also by the number of studies in which associations have been replicated, and the evolutionary context of the associated mutations. Therefore, e-GRASP will be a valuable resource for aiding researchers in the identification of bona fide, reproducible genetic associations from GWAS results. This resource is freely available at http://www.mypeg.info/egrasp.

## Background

In genome-wide association studies (GWAS), tens of thousands to millions of genomic loci are genotyped across large population samples of disease (case) and healthy (control) individuals to identify genetic variation that is associated with the presence of a disease trait. Similarly, continuous disease-related traits are also tested for association with genetic variation in large population samples of individuals with trait variation. An estimate of effect size (odds ratio or coefficient) and statistical significance (*P*-value) of association is determined for each tested locus. Results are typically ordered by *P*-value, and a multiple hypothesis adjusted *P*-value threshold (e.g., < 5 × 10^−8^) is often used to determine genome-wide significant associations. To date, thousands of putative trait-associated genetic variants (dSNPs) underlying complex disease have been identified [1], the majority of which are annotated in the GRASP2 resource [2]. However, discovered dSNPs vary among studies and they often explain relatively small fractions of the total heritability of the respective disease trait [3].

Here, we have added an evolutionary dimension to the GRASP2 database. We have integrated this resource with evolutionary information that complements GWAS results by contributing orthogonal predictions of dSNP functional importance. In particular, dSNPs located at evolutionarily conserved positions (due to lower evolutionary rates of change and greater inter-species evolutionary time span) are more likely to be functionally important or contribute to functional disruption and, thereby, potentially causally related to the associated phenotype(s). For example, using published data from a large case-control study of several common disease traits [4], we have recently shown that E-rank incorporating evolutionary conservation scores and the allelic *P*-value of phenotypic association enhances the discovery of alleles with a greater probability of bona fide and reproducible genetic disease associations, many of which may explain greater heritability [5].

In the following, we describe e-GRASP, which is a web resource to enable researchers to easily explore dSNPs present in the GRASP2 database by statistical significance of association, number of studies in which an association was replicated, and the evolutionary context in which the SNP occurred. We anticipate that this integrated resource will expedite the discovery of biologically meaningful genotype-phenotype associations from genome-wide association studies (GWAS) of human disease.

## Construction and content

### Data sources

The current version of the e-GRASP resource includes phenotype association measures of significance (*P*-values) and population allele frequency data for SNPs present in the GRASP2 database [2], which provides information on ~8.87 million SNPs-phenotype associations with a statistical significance threshold of ≤ 0.05 aggregated from 2082 Genome-Wide Association Studies that span 177 broad phenotypic categories. To date, this resource is the largest and most comprehensive publically available GWAS database [2]. Moreover, we will check for future updates to GRASP2 no less than two times per year and update e-GRASP accordingly.

We obtained 46 species alignments available from UCSC’s genome browser [6] and applied evolutionary methods to calculate evolutionary rates and evolutionary timespans for each SNP using the mammalian subset (33 species). To annotate coding mutations (mutations that occur within codons) with predictions of functional impact, we retrieved Polyphen2 [7] and SIFT [8] scores from the dbNSFP database [9]. For these SNPs, we also added functional impact scores produced by the EvoD method [10], which uses evolutionary-stratified predictive modelling and may provide different mutational diagnoses than Polyphen-2 and SIFT [10, 11].

### Evolutionary rate

For each SNP, we started with an alignment of 33 placental mammals obtained from the UCSC Genome Browser resource [6]. We estimated the evolutionary rate, Evol_Rate, of change at each site by dividing the total number of nucleotide substitutions in the phylogeny by the total time elapsed on the tree (substitutions per site per billion years) [5, 12].

### Evolutionary timespan

For each SNP, we again started with an alignment of 33 placental mammals obtained from the UCSC Genome Browser resource [6]. Evolutionary timespan, Evol_Span, quantifies the fraction of evolutionary time among species for which a given human nucleotide position has existed in the evolutionary history of the lineages in the phylogeny [5, 12] and, thus, its functional importance.

### E-rank

The E-rank method incorporates position-specific evolutionary measures to adjust GWAS *P*-values post hoc using independent prior evolutionary expectations. The power to detect true positive genetic associations in any individual study will depend on the study sample size and chosen significance threshold, as well as the variance and effect size of each locus [13]. The genetic variance at any SNP locus, reflected in population allele frequency, has a strong relationship with the evolutionary constraint at the position measured across species [14]. However, position-specific estimates of evolutionary constraint, such as evolutionary time span and evolutionary rate, are agnostic to variation segregating in contemporary humans and can be used as independent priors in the functional evaluation of modern human genetic variation [15]. Therefore, we use Evol_Span and Evol_Rate of each GWAS tested SNP allele and the corresponding minor allele frequency (MAF) to calculate the evolutionarily adjusted allelic association *P*-value rank, E-rank, as previously described [5]. Briefly, E-rank = (P/MAF) × (1/[K_r_ · K_t_]), where P is the phenotype-association P-value of the position, K_r_ is the rank of the Evol_Rate of the position and K_t_ is the rank of the Evol_Span of the position.

### Database architecture and implementation details

**Fig. 1:**
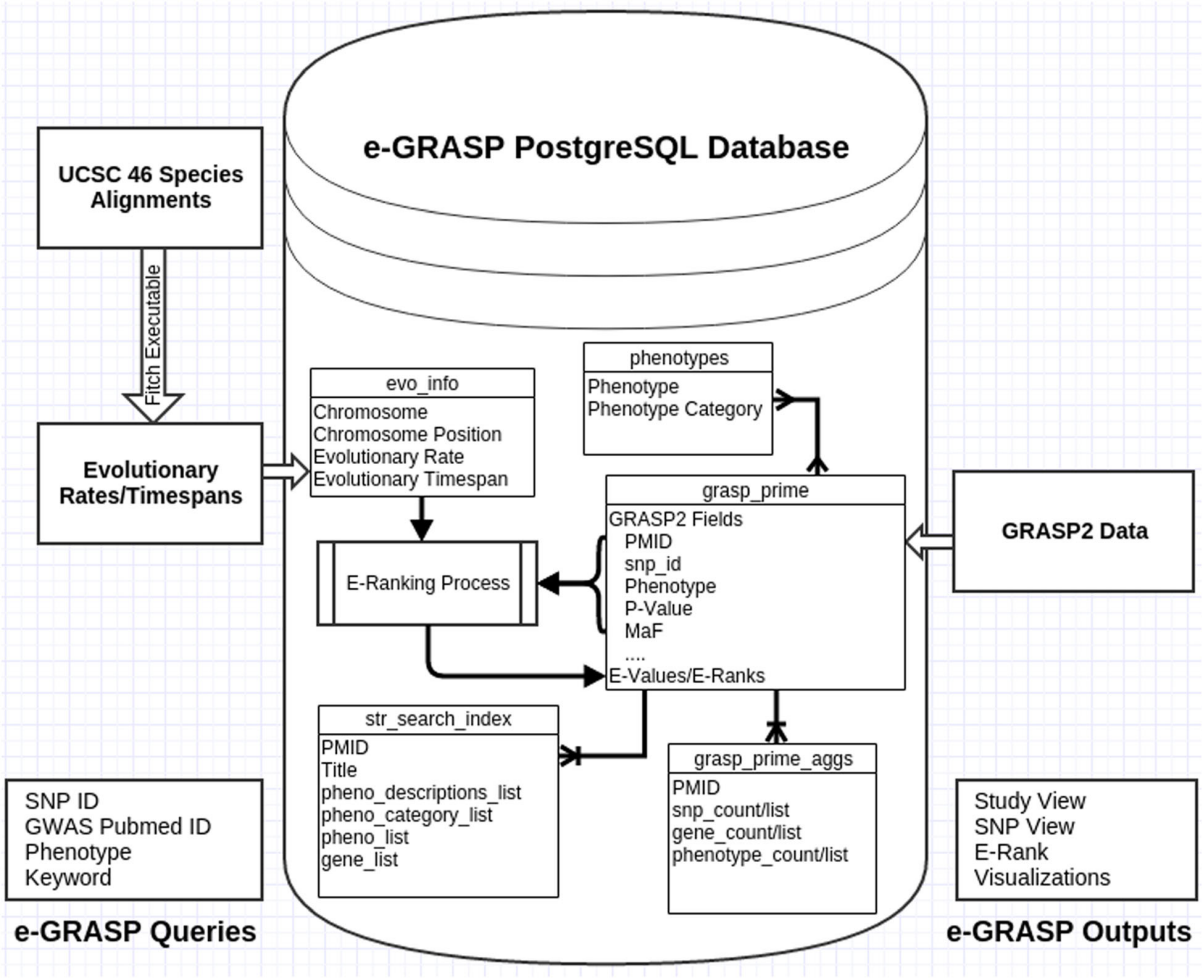
e-GRASP data flow and framework. e-GRASP includes external information from the GRASP2 database and evolutionary rates and times calculated from a multiple species alignment (indicated by *open arrows*). Internal interactions are indicated with *black arrows*. Searching may occur by SNP ID (rsid), GWAS Pubmed ID (PMID), phenotype, or keyword

We use a PostgreSQL database back-end to store and query the GRASP2 trait association data along with information about each mutation’s evolutionary context. One advantage of storing the GRASP2 data is that it improves query processing time. Although the bulk of the data is stored in a single table indexed by SNP id (rsid) and Pubmed citation id (PMID), there are a number of helper tables designed to speed up aggregation and searching by keyword or by phenotype and phenotype category (Fig. 1). The e-GRASP web-interface is written in PHP, making use of the jQuery JavaScript library to retrieve queried data from the database and display the results.

## Utility and discussion

### Search interface

The e-GRASP home page (http://www.mypeg.info/egrasp) contains a description of the resource, citation information and a navigation link to the search page. The search page includes the option to search by single SNP, multiple SNPs, single PMID, multiple PMIDs, phenotype, or keyword.

### Single SNPs

**Fig. 2:**
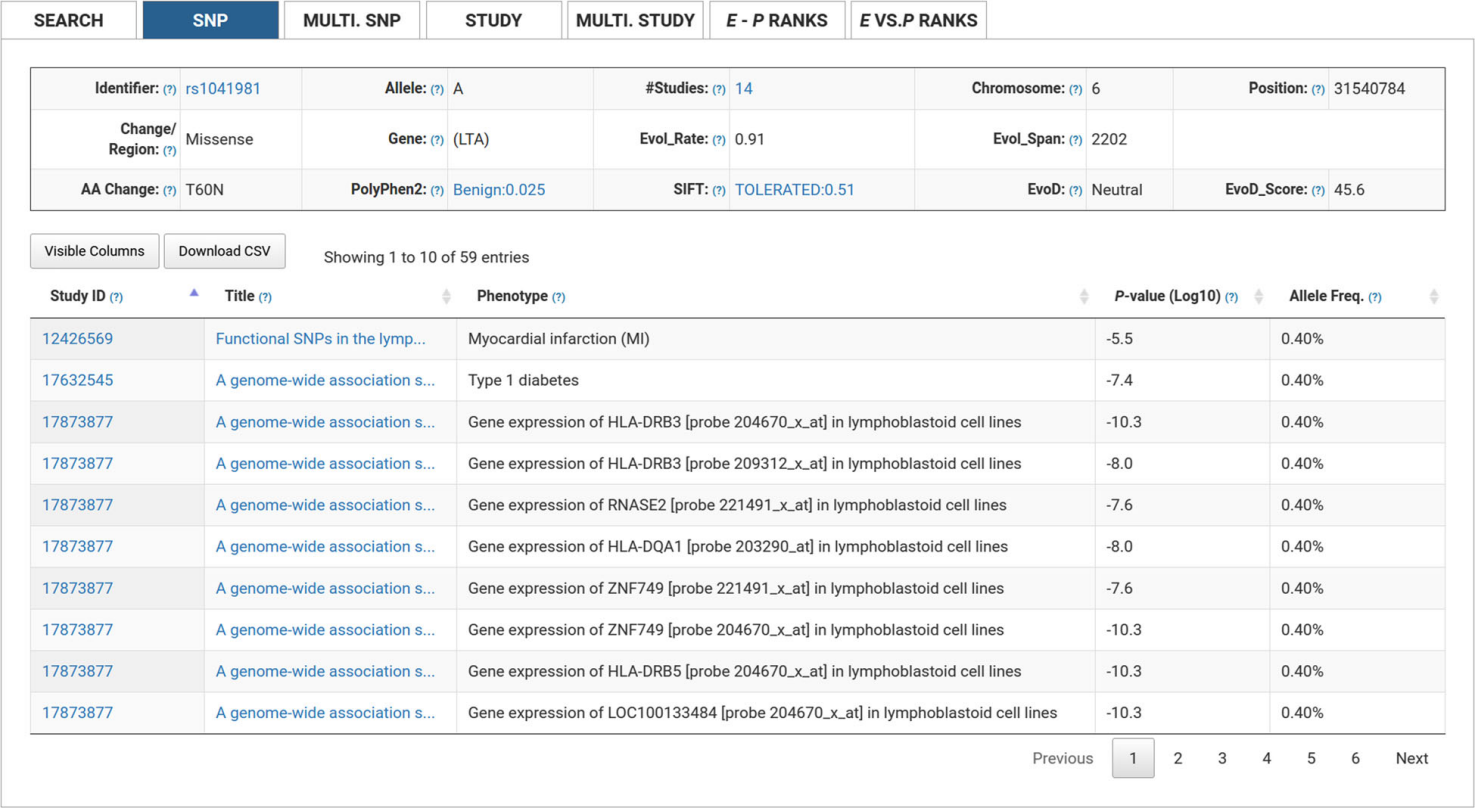
SNP View. Users can retrieve results for a single SNP, including summary data for that SNP (e.g. the number of studies the SNP is present in, the chromosome and chromosome position of the SNP, and SNP type), evolutionary prediction data (e.g. Evol_Rate, Evol_Span, PolyPhen2, SIFT, EVOD), as well as a table of all GWAS associations for that SNP in the GRASP2 database (including Study ID, Study Title, Phenotype, *P*-value (Log10), and allele frequency)

Searching for a single SNP will produce summary data for that SNP (Fig. 2), including general information (allele, allele type, chromosome, chromosome position (B37, hg19), number of included GWAS, and if applicable, amino acid change and gene), evolutionary context (evolutionary rate and evolutionary span), coding sequence impact (if applicable, PolyPhen2, SIFT, EvoD), as well as a table displaying data for each study/phenotype the SNP is associated with in GRASP2 (*P*-value ≤ 0.05). Users may also choose to download tabulated e-GRASP query results. Users may sort single-SNP query results by: [Study ID] PMID or [Title] the title of the paper containing the SNP, [Phenotype] the phenotype associated with the SNP, [*P*-value (Log10)] the log10 *P*-value of the SNP/phenotype association test, or [Allele Freq.] the MAF.

For missense mutations, e-GRASP also provides allelic tolerance predictions from SIFT, which uses a sequence homology-based approach, and disease-impact predictions from Polyphen2 which uses sequence and structure-based features. Both datasets are sourced from the dbNSFP database publicly available at https://sites.google.com/site/jpopgen/dbNSFP [3]. For most missense mutations, we also provide our own disease diagnoses and impact scores produced by the EvoD method [10]. EvoD is an alignment-based method for predicting the impact of missense mutations, which uses a separate model for each of three potential SNP classifications, ultra-conserved, well-conserved, or less-conserved, based on the site’s evolutionary rate being zero, between zero and one, or greater than one, respectively.

The example presented in Fig. 2 shows rs1041981, which has been implicated in multiple GWAS phenotypes, including Type 1 diabetes and myocardial infarction. Log10 *P*-values range from −10.3– −5.5, and functional predictions are consistent across PolyPhen, Sift and EvoD (benign, tolerated, neutral, respectively).

### Multiple SNPs

**Fig. 3:**
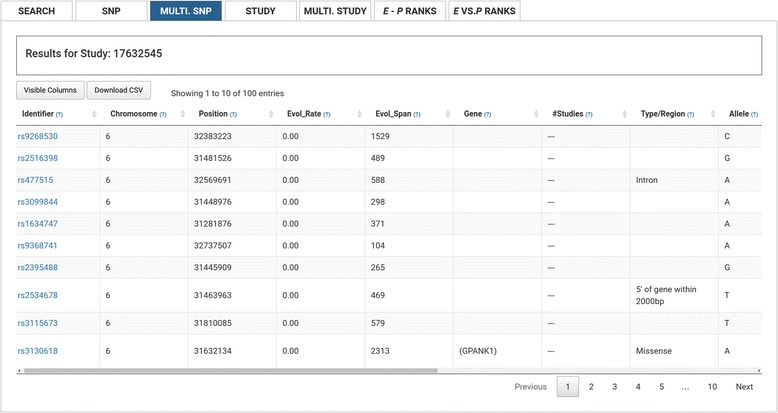
Multi SNP View. Users can retrieve a table of summary data (e.g., chromosome and chromosome position of the SNP, which if any gene the SNP is located in, the number of studies the SNP is present in, SNP type) for each SNP within a given study

Multiple SNP searches return a table of summary data for each SNP (Fig. 3), including rsid, chromosome, chromosome position (B37, hg19), evolutionary rate, evolutionary span, genic location, the number of GRASP2 studies and phenotypes each tested SNP generated a *P*-value ≤ 0.05, region type (e.g., intron, missense, 3′UTR), the *P*-value range across GRASP2 studies, the SNP allele corresponding to the evolutionary rate, evolutionary span and MAF, and for the subset of missense SNPs, the amino acid change, and Polyphen2 and SIFT scores. The Fig. 3 example displays a subset of these estimates for a set of SNPs located on chromosome 6, including one missense SNP.

Multi-SNP results may be sorted by: [Identifier] rsid, [Chromosome] the chromosome the SNP is located on, [Position] the chromosomal position of the SNP, [Evol_Rate] the evolutionary rate of the SNP, [Evol_Span], the evolutionary time span of the SNP, [In Gene] the gene(s) in which the SNP is located, [Number of Studies] the number of distinct GWAS papers in which the SNP is found, [Number of Phenotypes] the number of distinct phenotypes associated (*P*-value ≤ 0.05) with the SNP, [Min/Max *P*-value] the minimum/maximum *P*-value associated with the SNP across all studies and phenotypes, [Type/Region] the category of genomic region in which the SNP occurs region (e.g., intron, missense, 3′UTR), [Allele] the GWAS minor allele, [Allele Freq.] the MAF, [AA Change] the coding sequence consequence of the SNP, [PolyPhen2], the PolyPhen score, or [Sift], the Sift score.

### Study view filters

**Fig. 4:**
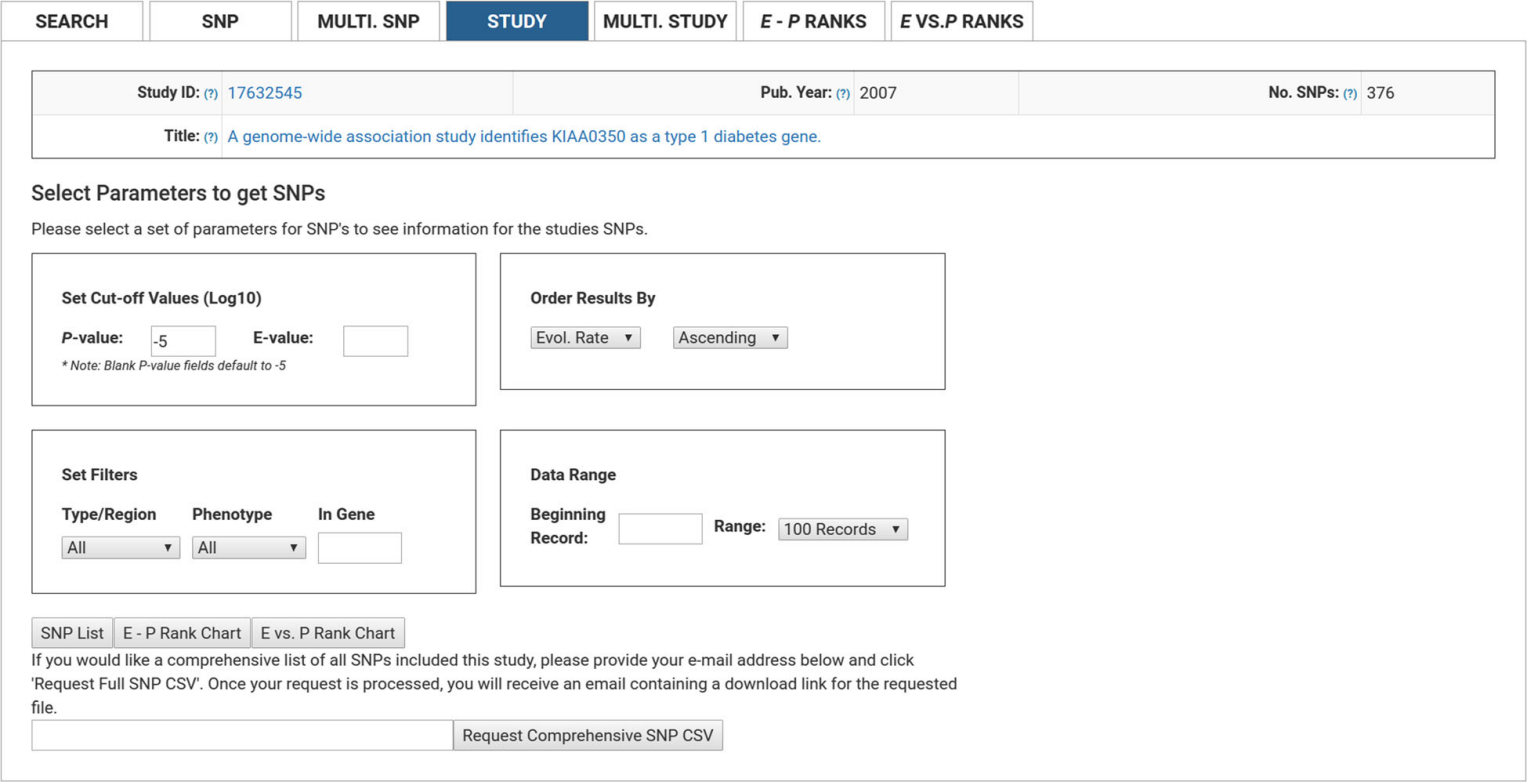
Study View Filter. Users can filter results for a given study to only include associations which exceed a given threshold of statistical significance (e.g., *P-value* (Log10) ≤ −5), or to only include SNPs within a specified gene, associated with a specified phenotype, or filtered by SNP type

e-GRASP searches can include one or more studies (by PMID) and offers the following SNP filter parameters (Fig. 4): *P*-value cutoff, *E*-value cutoff, SNP type (e.g., intron, missense, 3′UTR), phenotype, or by a particular gene.

Study query results containing multiple SNPs may be sorted by the same criteria that multiple SNP query results use (listed above; Fig. 3), and multiple study queries may be sorted by: [Pubmed ID] PMID or [Title] the title of the paper containing the SNP, [No. SNPs] the number of SNPs from a given study with a *P*-value ≤ 0.05, [No. Phenotype] the number of phenotypes that were tested in the study, or [No. Genes], the number of genes containing SNPs from a given study with a *P*-value ≤ 0.05.

### e-GRASP SNP rankings

**Fig. 5:**
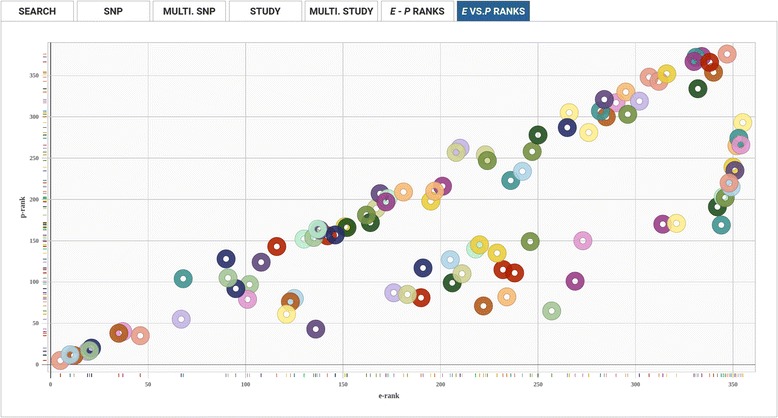
E- vs P-Ranks Visualization. Allows users to visualize the relationship between the statistical significance (displayed on the y-axis) and evolutionary-adjusted statistical significance (displayed on the x-axis) of SNPs, in a given paper, as a scatterplot

**Fig. 6:**
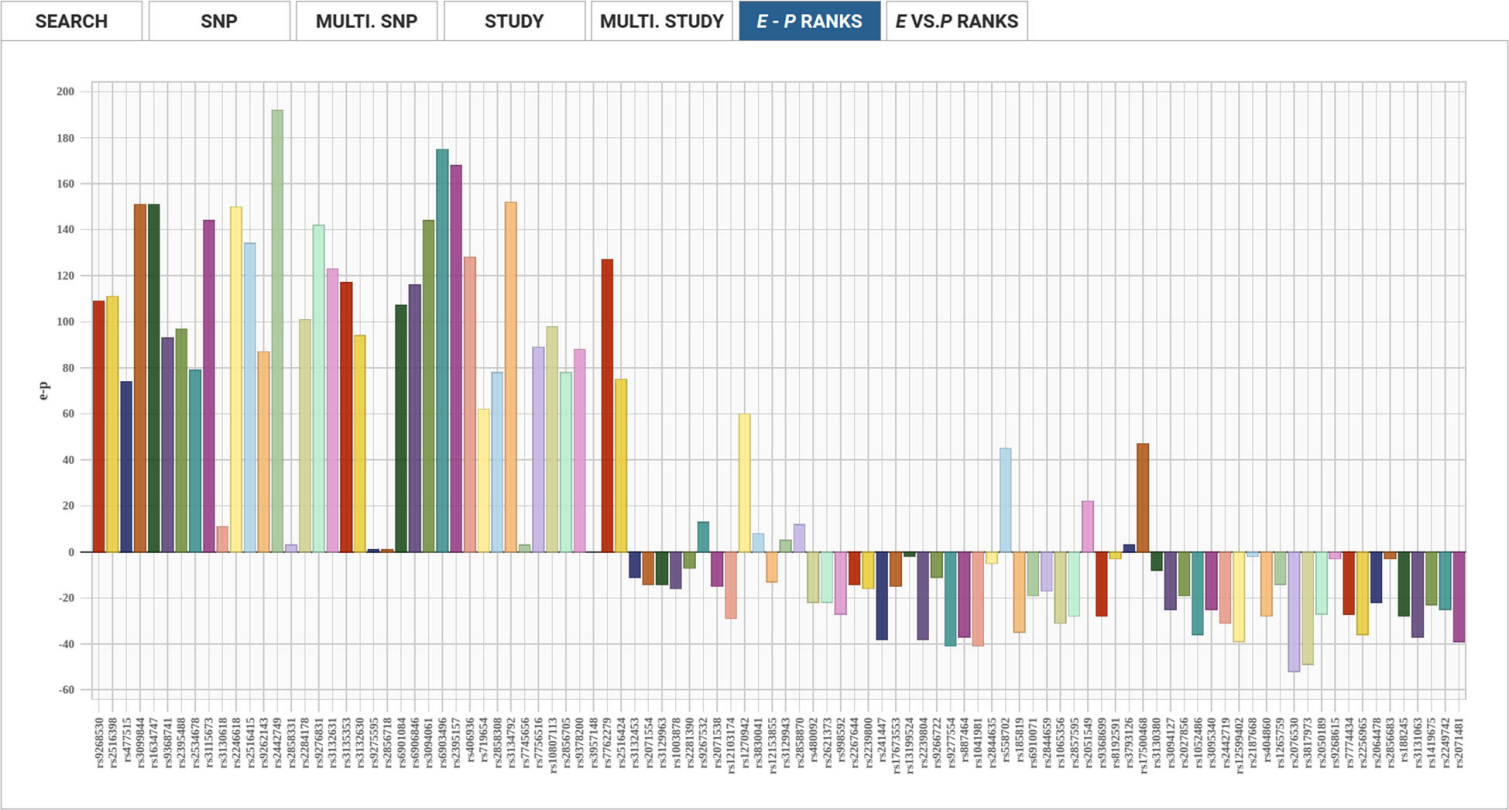
E-P Ranks Visualization. Visually highlights SNPs in a paper whose relative significance is most enhanced by evolutionary adjustment. The x-axis displays each SNP, and the y-axis displays E-rank minus P-rank

The e-GRASP interface also provides two outputs for visually comparing the P-Rank (rank by phenotype-association *P*-value) of SNPs within a study to their E-Rank: E-Rank vs. P-Rank and E-Rank – P-Rank, (Figs. 5 and 6, respectively). When the user hovers the curser over individual SNP points (e.g. Fig. 5) or plot bars (e.g. Fig. 6), a details bubble pops up with additional SNP-specific information, including the rsid and plotted values. In addition, preemptive sorting of multiple SNPs from a given study can be used to adjust the order that SNPs appear in the E-Rank/P-Rank plots. While the web-based tabulated and plotted results can include up to 1000 SNPs to ensure that the corresponding E-Rank/P-Rank plots are interpretable, preemptive sorting allows the user to view the results deemed most important. Users may choose to additionally bulk download all results into a CSV file without any limit to the number of included SNPs.

## Conclusions

By allowing users to browse the GRASP2 data in conjunction with easily sortable measures of SNP replication as well as evolutionary context, the e-GRASP resource enables researchers to more easily find highly reproducible statistically significant SNPs with genuine phenotypic associations within GWAS results from GRASP2 and integrate this information with evolutionary information. e-GRASP also provides tools for visually comparing the raw statistical trait-association significance of a SNP with its evolutionary-adjusted significance, in order to highlight SNPs whose biological significance is clarified when taking into account position-specific evolutionary context.

## Abbreviations

dSNPs: Disease-associated genetic variants
E-rank: Evolutionary-adjusted SNP ranking
Evol_Rate: Evolutionary rate
Evol_Span: Evolutionary time span
GRASP2: Genome-wide repository of Associations between SNPs and phenotypes
GWAS: Genome-wide association studies
MAF: Minor allele frequency
PMID: Pubmed ID
RSIDs: SNP IDs
SNPs: Single nucleotide polymorphisms
