# Erratum to: e-GRASP: an integrated evolutionary and GRASP resource for exploring disease associations

**DOI:** 10.1186/s12864-017-3647-0

**Published:** 2017-04-06

**Authors:** Sajjad Karim, Hend Fakhri Nour Eldin, Heba Abusamra, Nada Salem, Elham Alhathli, Joel Dudley, Max Sanderford, Laura B. Scheinfeldt, Adeel G. Chaudhary, Mohammed H. Al-Qahtani, Sudhir Kumar

**Affiliations:** 1 Center for Excellence in Genome Medicine and Research, King Abdulaziz University, Jeddah, Saudi Arabia; 2 Department of Genetics and Genomic Sciences, Mount Sinai School of Medicine, New York, NY 10029 USA; 3 Institute for Genomics and Evolutionary Medicine, Temple University, Philadelphia, PA 19122 USA; 4 Department of Biology, Temple University, Philadelphia, PA 19122 USA

## Erratum

This article [1] unfortunately published with an author deleted in the author list. The correct author list is presented above.

The author list was previously incorrectly presented as:

Sajjad Karim^1^, Hend Fakhri NourEldin^1^, Heba Abusamra^1^, Nada Salem^1^, Elham Alhathli^1^, Joel Dudley^2^, Max Sanderford^3^, Laura B. Scheinfeldt^3,4^ and Sudhir Kumar^1,3,4*^

^1^Center for Excellence in Genome Medicine and Research, King Abdulaziz University, Jeddah, Saudi Arabia. ^2^Department of Genetics and Genomic Sciences, Mount Sinai School of Medicine, New York, NY 10029, USA. ^3^Institute for Genomics and Evolutionary Medicine, Temple University, Philadelphia, PA 19122, USA. ^4^Department of Biology, Temple University, Philadelphia, PA 19122, USA.
